# Supplementary material for: EGFL9 promotes breast cancer metastasis by inducing cMET activation and metabolic reprogramming
Source: Nat Commun. 2019 Nov 6;10:5033. doi: 10.1038/s41467-019-13034-3 (PMC6834558; doi:10.1038/s41467-019-13034-3)
Supplement: Supplementary file 1 — Supplementary Information [file 41467_2019_13034_MOESM1_ESM.pdf]

Supplementary Information

EGFL9 Promotes Breast Cancer Metastasis by Inducing cMET Activation and Metabolic Reprogramming

Meng et al.

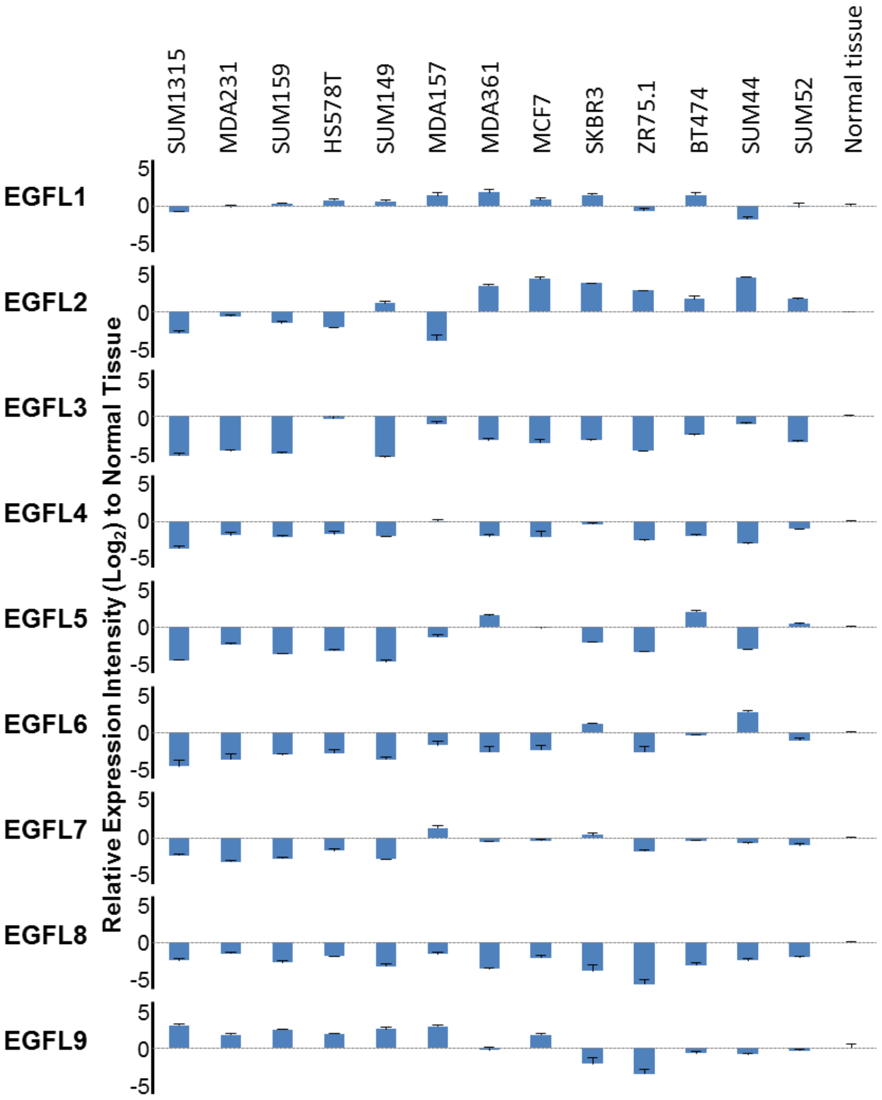

Supplementary Figure 1. Relative expression of EGF like protein family members in human breast cancer cell lines. Real time PCR examination of nine EGF-like protein family members expression in 14 human breast cancer cell lines. The 14 human breast cancer cell lines are 1:SUM1315; 2: MDA -MB 435; 3:MDA -MB 231; 4: SUM159; 5:HS578T; 6: SUM149; 7:MDA-MB 157; 8: MDA -MB 361; 9:MCF7; 10: SKBR3; 11: ZR75.1; 12: BT474; 13: SUM44; 14:SUM52. Eight of these cell lines (from 1 to 8) are basal-like breast cancer cell lines and the other five (from 9 to 14) are luminal cell models.

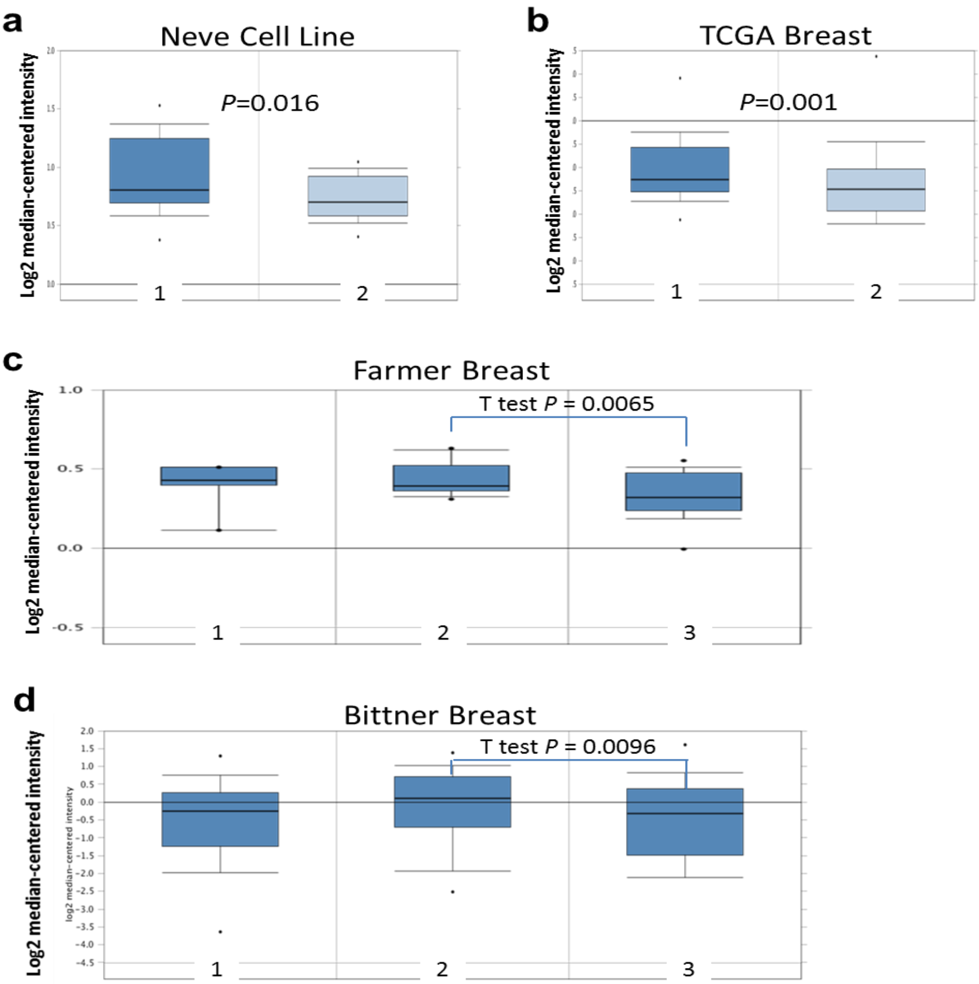

Supplementary Figure 2. Analysis of EGFL9 expression in TNBC cells and tumors based on datasets in Oncomine. (a) EGFL9 expression is higher in TNBC cell lines than in non-TNBC cell lines. *P*=0.016. The data link is: http://www.ebi.ac.uk/arrayexpress/experiments/E-TABM-157. 1: TNBC cell lines (n=21); 2: Non-TNBC cell lines (n=25). (b) EGFL9 expression is higher in TNBC tumor samples than in non-TNBC tumor samples. *P*=0.001. The data link is: http://tcga-data.nci.nih.gov/tcga/. 1: TNBC tumors (n=46); 2: Non-TNBC tumors (n=250). (c) EGFL9 expression is higher in basal-like subtype of invasive breast carcinoma samples than in luminal-like subtype of invasive breast carcinoma samples. *P*=0.0065. The data link is: http://www.ncbi.nlm.nih.gov/geo/query/acc.cgi?acc=GSE1561. 1: Apocrine breast carcinoma (6); 2: basal-like subtype of invasive breast carcinoma samples (16); and 3: Luminal-like subtype of invasive breast carcinoma samples (n=27). (d) EGFL9 expression is higher in TNBC samples (n=45) than in non-TNBC samples (n=177). *P*=0.0096. The data link is: http://www.ncbi.nlm.nih.gov/geo/query/acc.cgi?acc=GSE2109. 1: No value (n=114); 2: TNBC tumors (n=45); and 3: Non-TNBC tumors (n=177). Two tailed unpaired *t*-test was used for comparing two groups of data. Statistical significance was considered at a value of *P*<0.05.

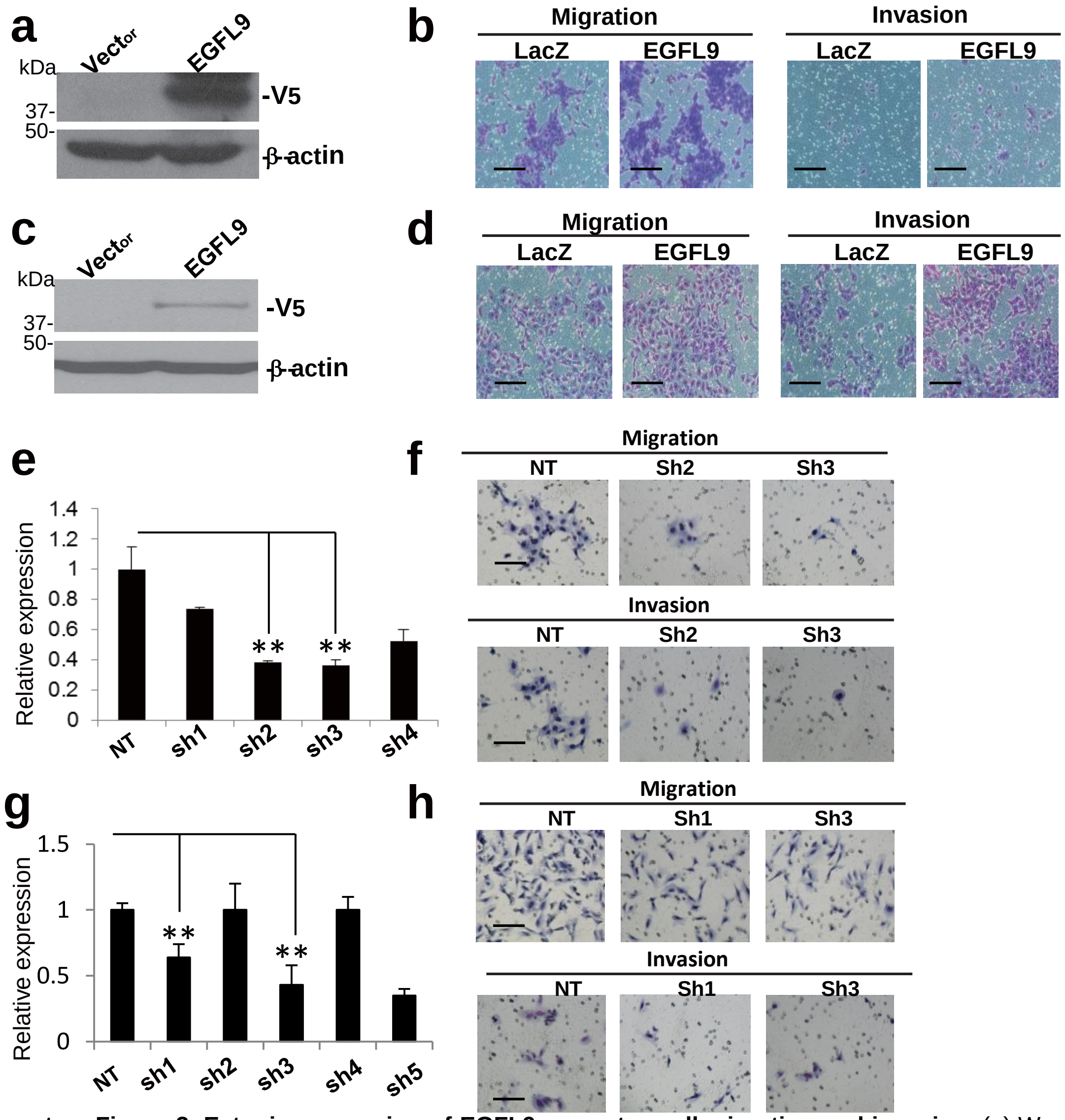

Supplementary Figure 3. Ectopic expression of EGFL9 promotes cell migration and invasion. (a) Western blot analysis showed ectopic expression of EGFL9 in HMLE cells. EGFL9 protein is detected with V5 antibody. β-actin was used as the protein loading control. (b) Representative figures show the chamber assay for cell migration (left panel) and invasion (right panel) in the HMLE cell line. Scale bar: 100 µm. (c) Western blot analysis showed ectopic expression of EGFL9 in EpRAS cells. EGFL9 protein is detected with V5 antibody. β-actin was used as protein loading control. (d) Representative figures show the chamber assay for cell migration (left panel) and invasion (right panel) in EpRas cell model. Scale bar: 100 µm. (e and g) Real time PCR analysis showed knockdown of EGFL9 in 4T1 (e) or SUM159 (g) cells. EGFL9 expression is detected with a real-time PCR analysis. Four clones showed 4 different shRNAs target EGFL9 in 4T1 cells. Five clones showed 5 different shRNAs target EGFL9 in SUM159 cells. NT: non-targeted control. Each bar represents the mean ± SD. for triplicate experiments. Unpaired two-tailed *t*-test was used for comparing two groups of data. For all experiments, * $P<0.05$; ** $P<0.01$; *** $P<0.001$. (f and h) Representative figures show the chamber assay for cell migration (left panel) and invasion (right panel) in 4T1 (f) and SUM159 (h) cells. Scale bar: 100 µm.

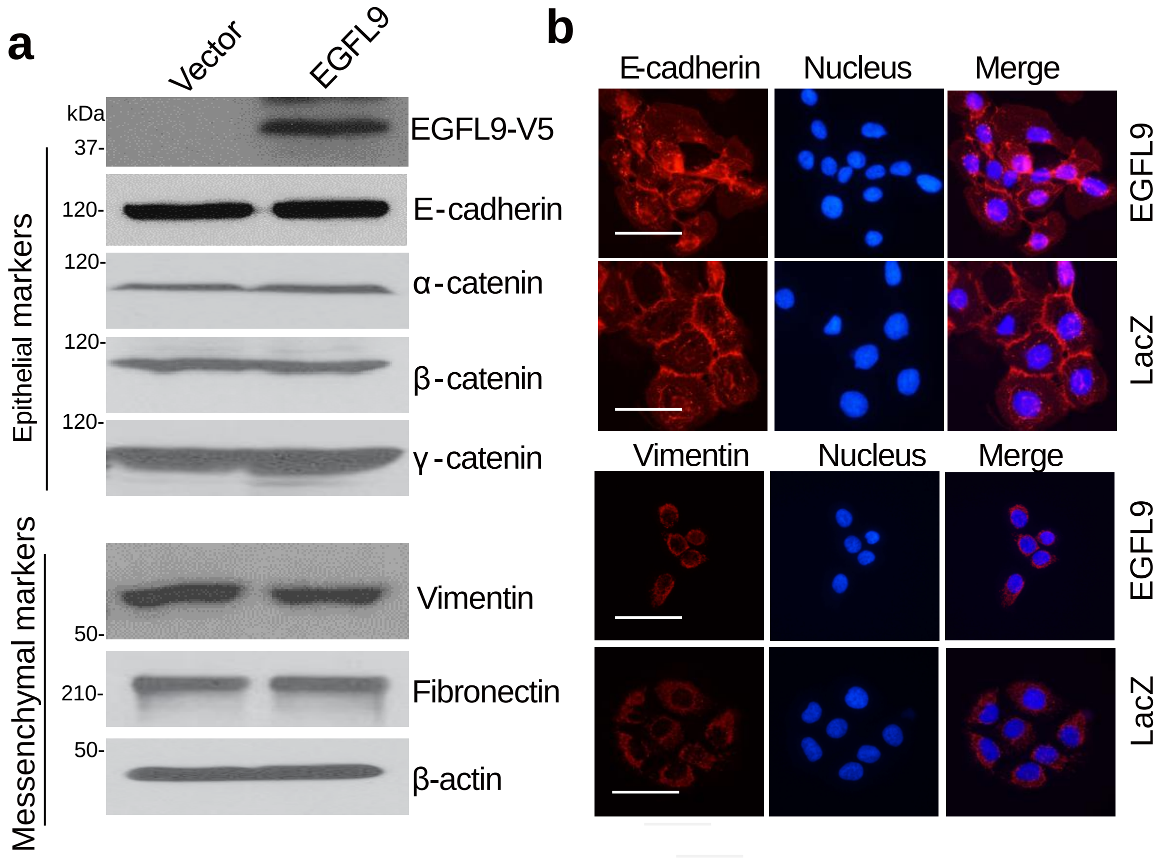

Supplementary Figure 4. The effect of EGFL9 on the EMT program in human mammary epithelial cells. (a) Western blotting demonstrates the effect of ectopic expression of EGFL9 on EMT markers. EGFL9 was detected by anti-V5 antibody. The other EMT markers were listed as indicated. (b) An immunofluorescence assay confirmed the results of the western blot in HMLE cells. The red signal represents the staining of the corresponding protein, and the blue signal represents the DAPI-stained nuclei. Scale bar: 50 μm.

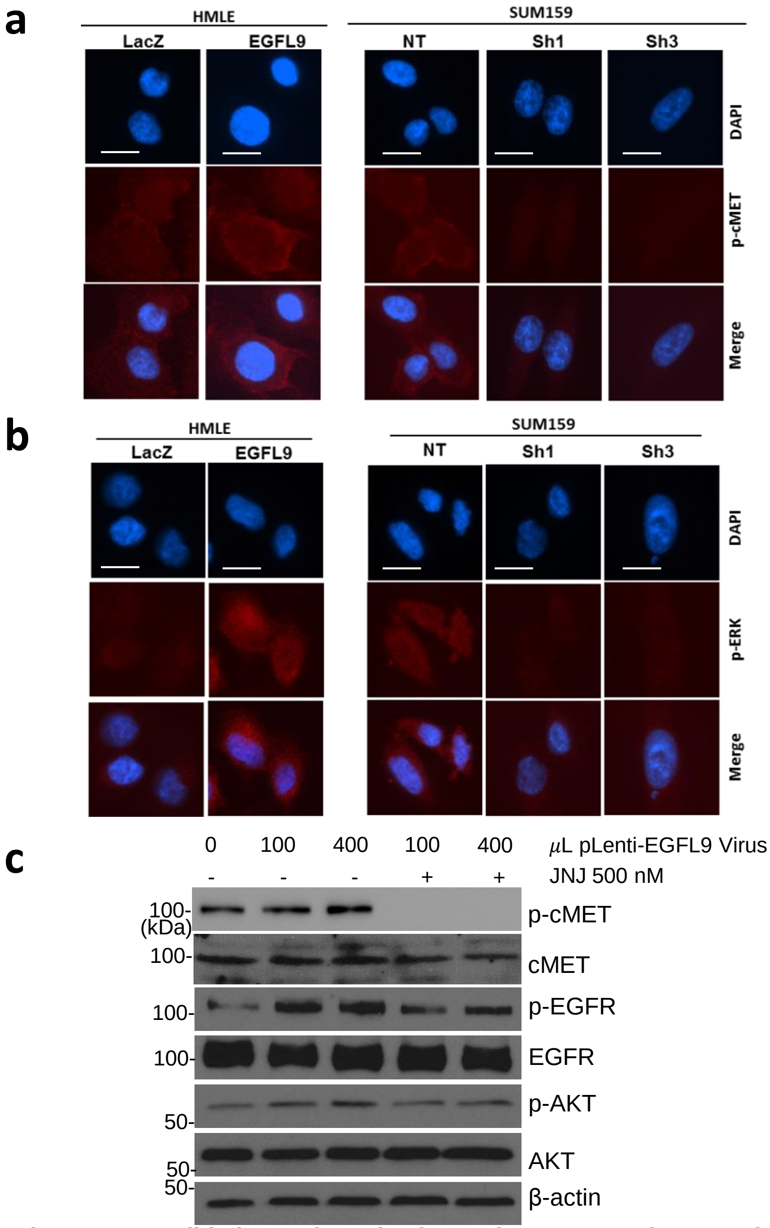

Supplementary Figure 5. Validation of activation of cMET and ERK by EGFL9. (a) An immunofluorescence assay confirmed the results of the western blots in HMLE cells and SUM159 cells. The red signal represents the staining of the p-cMET. Scale bar: 20 µm. (b) An immunofluorescence assay confirmed the results of the western blots in HMLE and SUM159 cells. The red signal represents staining for p-ERK. The blue signal represents the DAPI-stained nuclei. Scale bar: 20 µm. (c) Western blotting showed that targeting cMET activation will block EGFL9 to activate downstream signaling related to cancer metastasis. JNJ38877605 (500nM) was used to inhibit cMET activation as indicated. Different amounts of viruses expressing EGFL9 were added to cell culture medium to activate cMET signaling. Activation of pEGFR and pAKT was then examined by probe the phosphorylation of these proteins as critical molecules of the downstream signaling pathways.

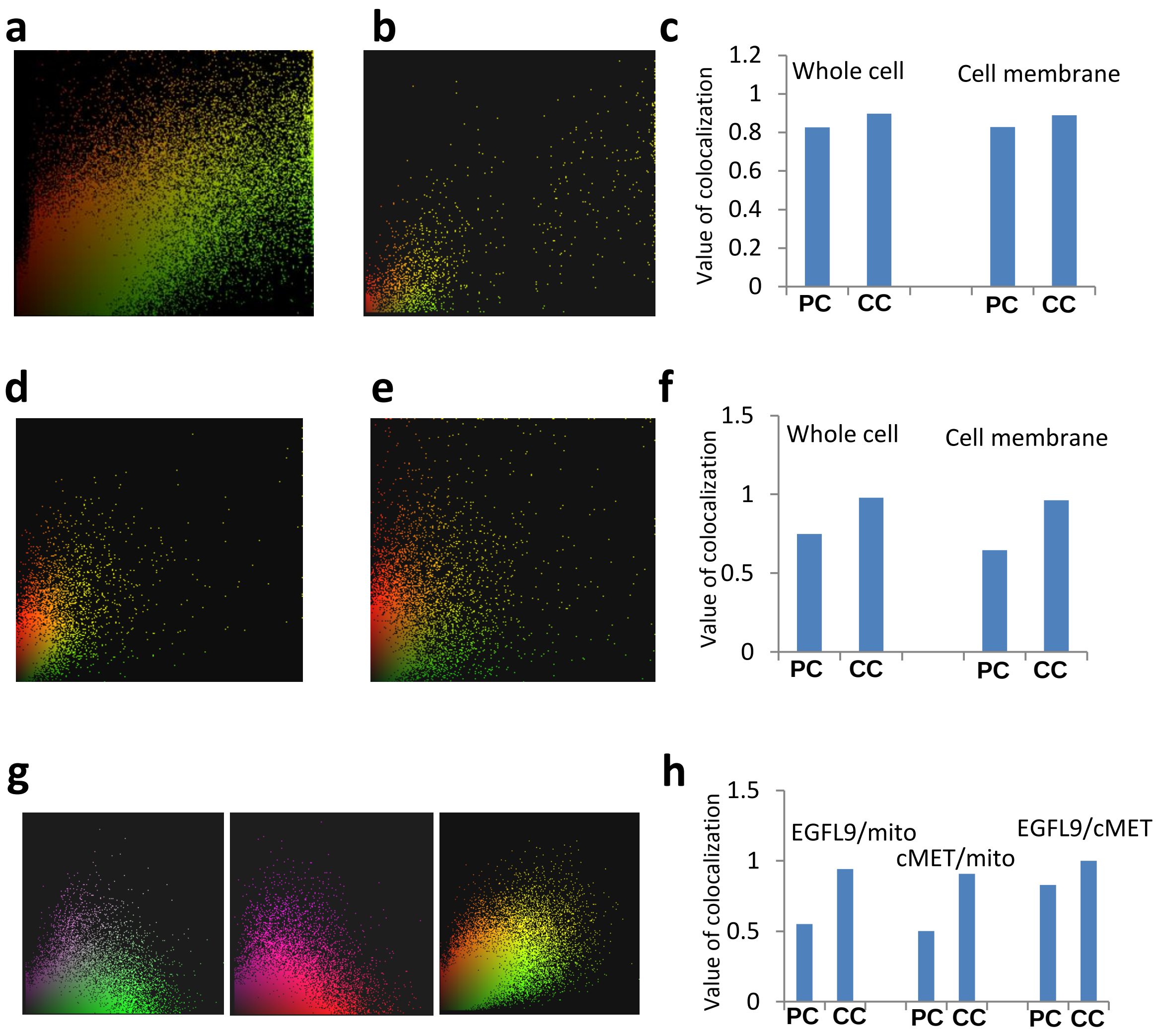

Supplementary Figure 6. Analyses of colocalization of EGFL9, cMET, and mitochondria. (a and b) Scatter plot of EGFL9 (green) and cMET (red) signaling in whole cell (a) and cell membrane (b) of HMLE/EGFL9 cells. (c) Quantitation of colocalization with Pearsons Correlation (PC) and Colocalization Coefficient M (CC). (d and e) Scatter plot of EGFL9 (green) and cMET (red) signaling in whole cell (d) and cell membrane (e) of SUM159 cells. (f) Quantitation of colocalization with Pearsons correlation (PC) and colocalization coefficient M (CC). (g) Scatter plot of EGFL9 (green)/Mitochondria (Purple) (left panel), cMET (red)/Mitochondria (purple) (middle panel), and EGFL9 (green)/cMET (red) (right panel) signals in HMLE/EGFL9 cells. (h) Quantitation of colocalization between EGFL9/Mito, cMET/Mito and EGFL9/cMET with Pearsons Correlation (PC) and Colocalization Coefficient M (CC).

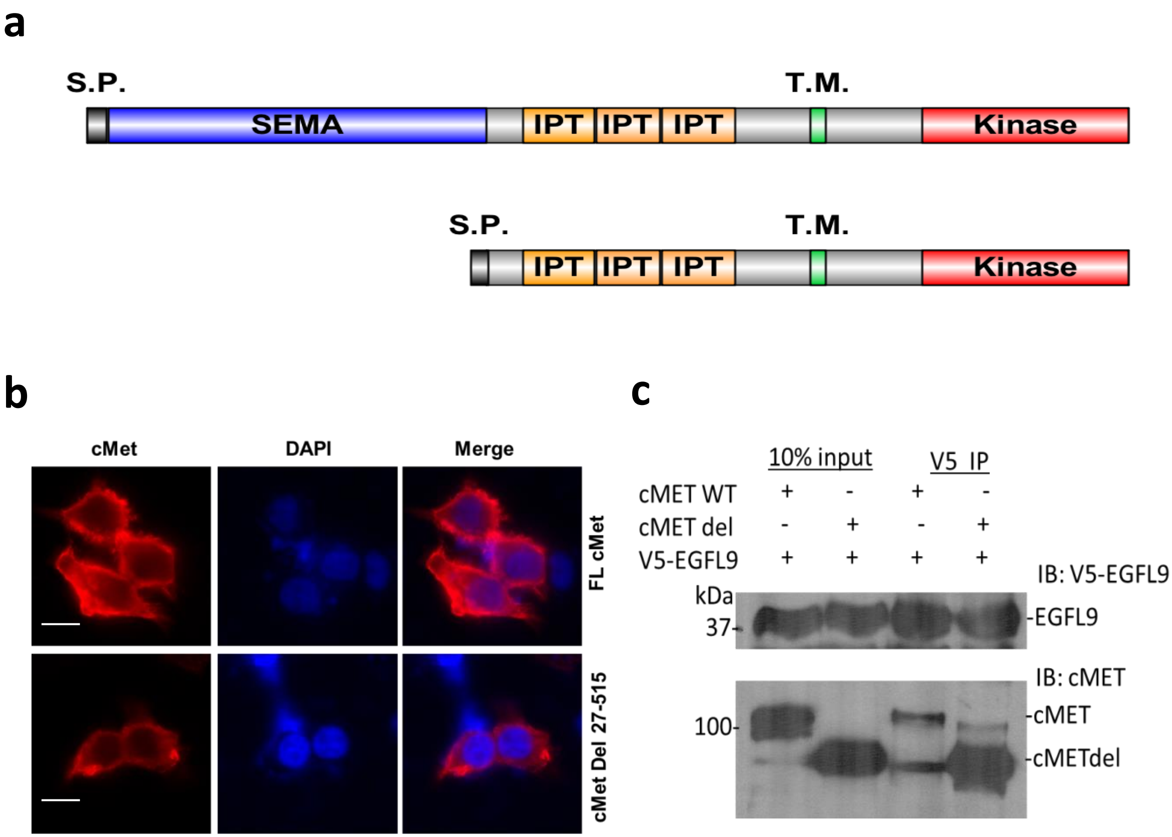

Supplementary Figure 7. cMET dimerization domain is not required for interaction with EGFL9. (a) Diagram of the structure of full-length cMET (FL-cMET) and cMET with deletion of the SEMA domain (cMETdel). S.P. stands for signaling peptides. T.M. stands for transmembrane domain. (b) Immunofluorescence assay showed the same cellular localization of FL-cMET and cMET with SEMA domain deletion in 293T cells. Scale bar: 20 µm. c) Interaction of EGFL9 and FL-cMET and cMET with SEMA domain deletion in 293T cells. EGFL9-V5 construct was co-transfected with FL-cMET or cMETdel, respectively, in 293T cells. Immunoprecipitation was performed with anti-V5 antibody followed by immunoblotting with anti-V5 (top panel) and anti-cMET (bottom panel) antibodies, respectively.

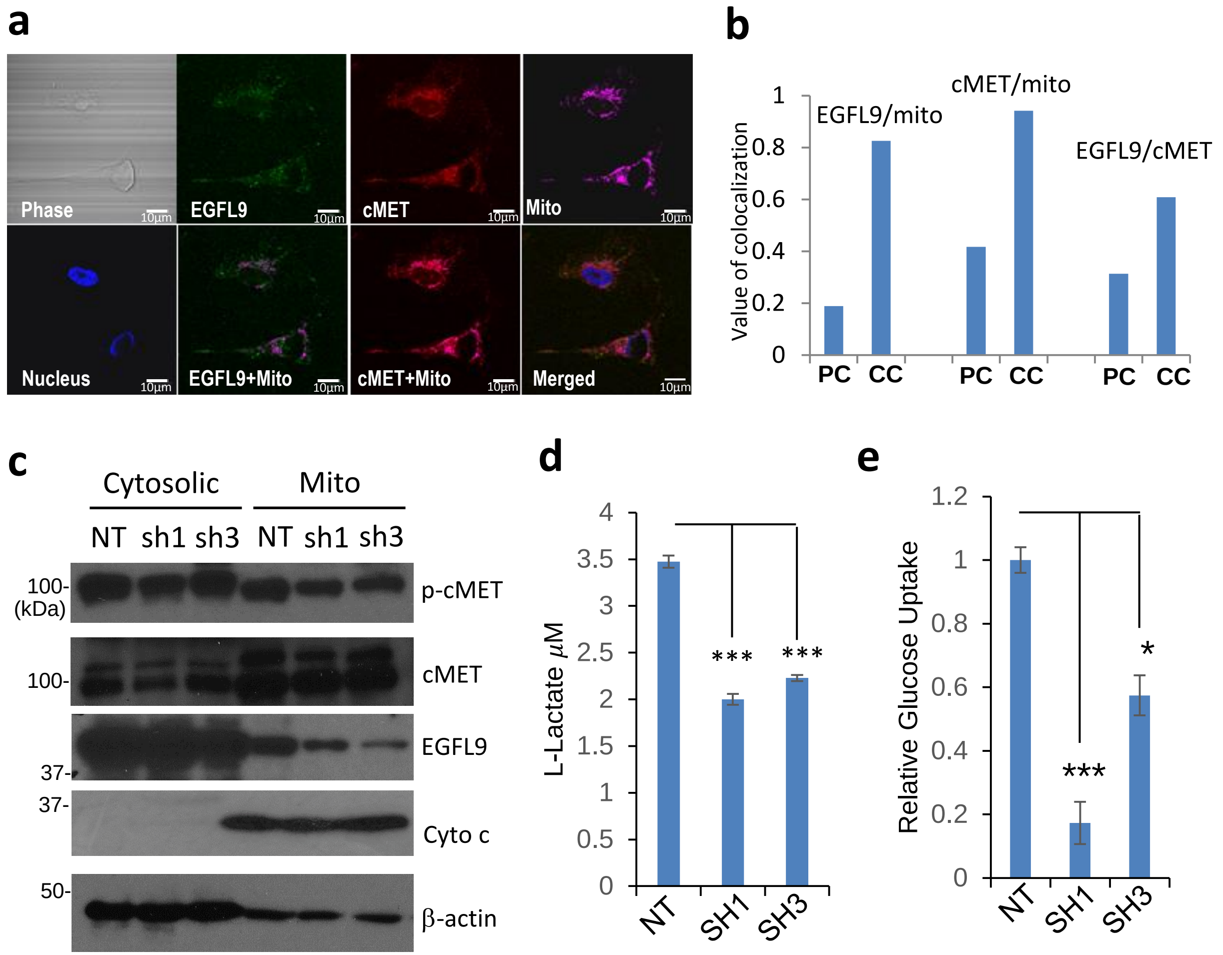

Supplementary Figure 8. EGFL9 and cMET colocalize in Mitochondria and its effect on metabolic switch. (a) Immunofluorescence assay shows colocalization of EGFL9 (green) and cMET (red) in the mitochondria (purple) in SUM159 cells. Scale bar: 10 µm. (b) Quantitation of colocalization EGFL9/Mito, cMET/Mito and EGFL9/cMET with Pearsons Correlation (PC) and Colocalization Coefficient M (CC). (c) Cytosolic and mitochondrial proteins were isolated from SUM159 cells w/wo EGFL9 knockdown and subjected to SDS–PAGE followed by probing with indicated antibodies. Cytochrome *c* is a mitochondrial marker. β-actin is a protein-loading control. (d) L-Lactate generation level in SUM159/NT, SUM159/EGFL9 sh1 and sh3 cell culture medium was measured using a glycolysis cell based assay. (e) The glucose concentration in SUM159/NT, SUM159/EGFL9 sh1 and sh3 cell culture medium was measured with the Amplex Red Glucose Assay Kit. For d and e panels, each bar represents the mean + SD. for triplicate experiments. Unpaired two-tailed *t*-test was used for comparing two groups of data. * $P<0.05$; ** $P<0.01$ and *** $P<0.001$.

a

b

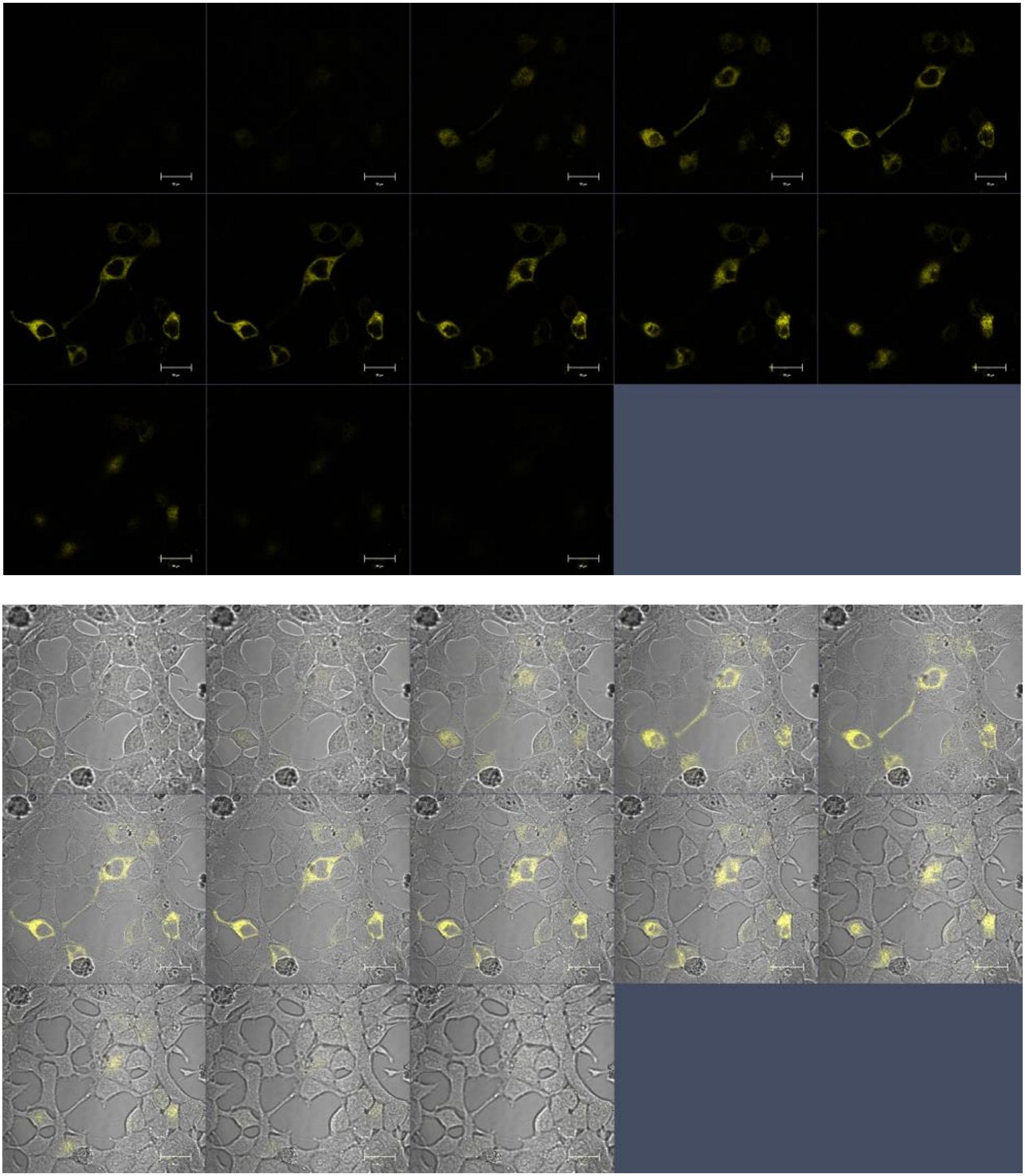

Supplementary Figure 9. The z-stack images for BiFC assay. (a) Z-stack images showed specific Venus fluorescence signals in 293T cells transfected with p3xFlag-CMV-EGFL9-VN and p3xFlag-CMV-COA3-VC. (b) Merged images of Venus fluorescence signals and bright field images of cells in 293T cells. Scale bar: 20 μm.

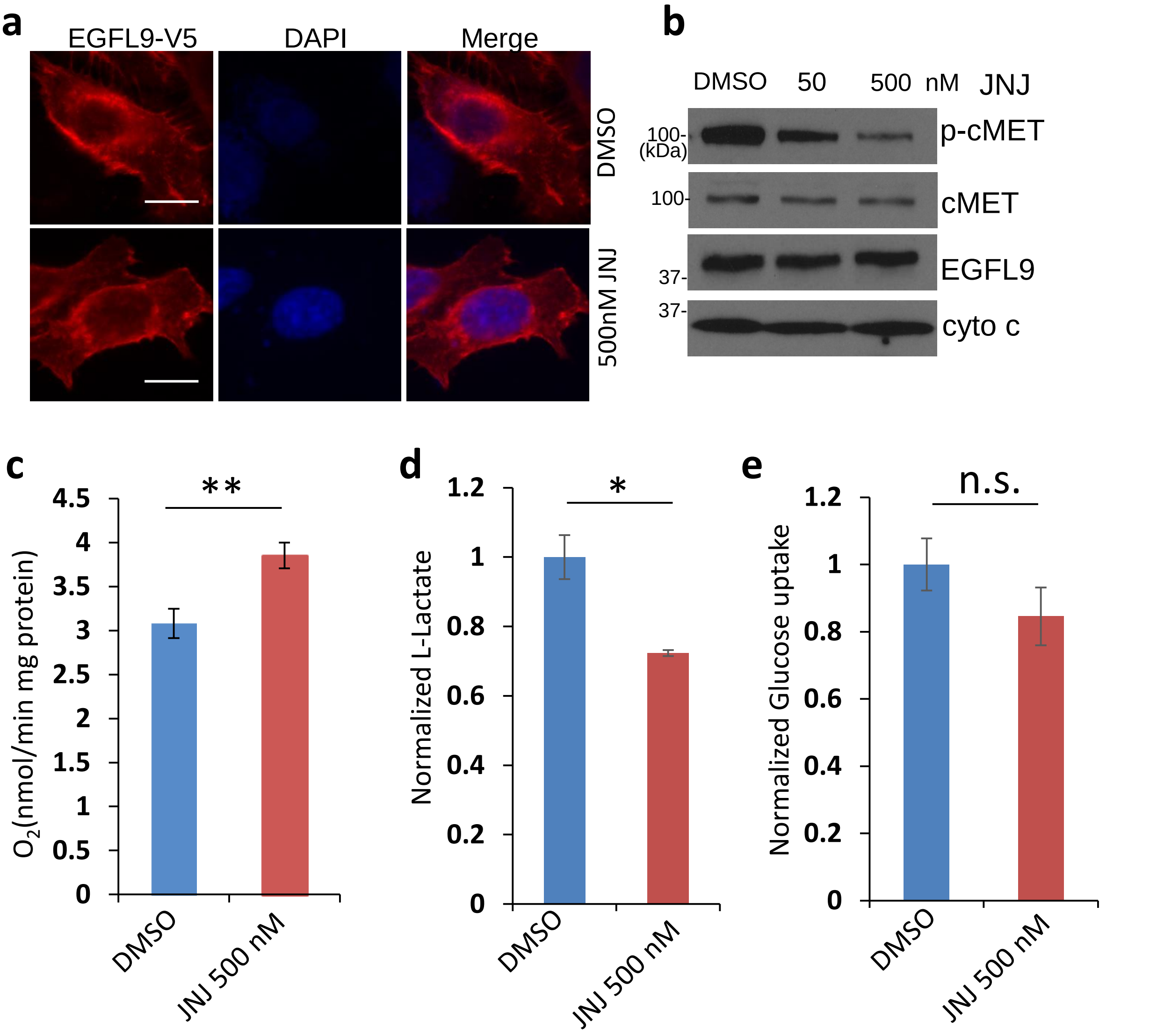

Supplementary Figure 10. The effect of cMET phosphorylation on EGFL9 driven metabolic changes. (a) EGFL9 subcellular distribution was not changed upon JNJ38877605 treatment as shown by an immunofluorescence assay. Scale bar: 20 µm. (b) Phosphorylation of cMET was significantly inhibited and EGFL9 expression was not changed due to JNJ38877605 treatment as shown by a Western blot analysis. (c) COX specific activity in HMLE/EGFL9 cells w/wo JNJ38877605 treatment (500 nM, 24 hr) was measured. (d) Lactate level in culture medium of HMLE/EGFL9 w/wo JNJ38877605 treatment (500 nM, 24 hr) was measured using a glycolysis cell based assay kit. (e) The glucose concentration in culture medium of HMLE/EGFL9 w/wo JNJ38877605 treatment (500 nM, 24 hr) was measured with the Amplex Red Glucose Assay Kit. For all three panels, each bar represents the mean ± SEM (standard error of the mean of a representative experiment performed in triplicate). *P*-value was determined by unpaired two-tailed *t*-test. N.S. $P>0.05$, * $P<0.05$, ** $P<0.01$.

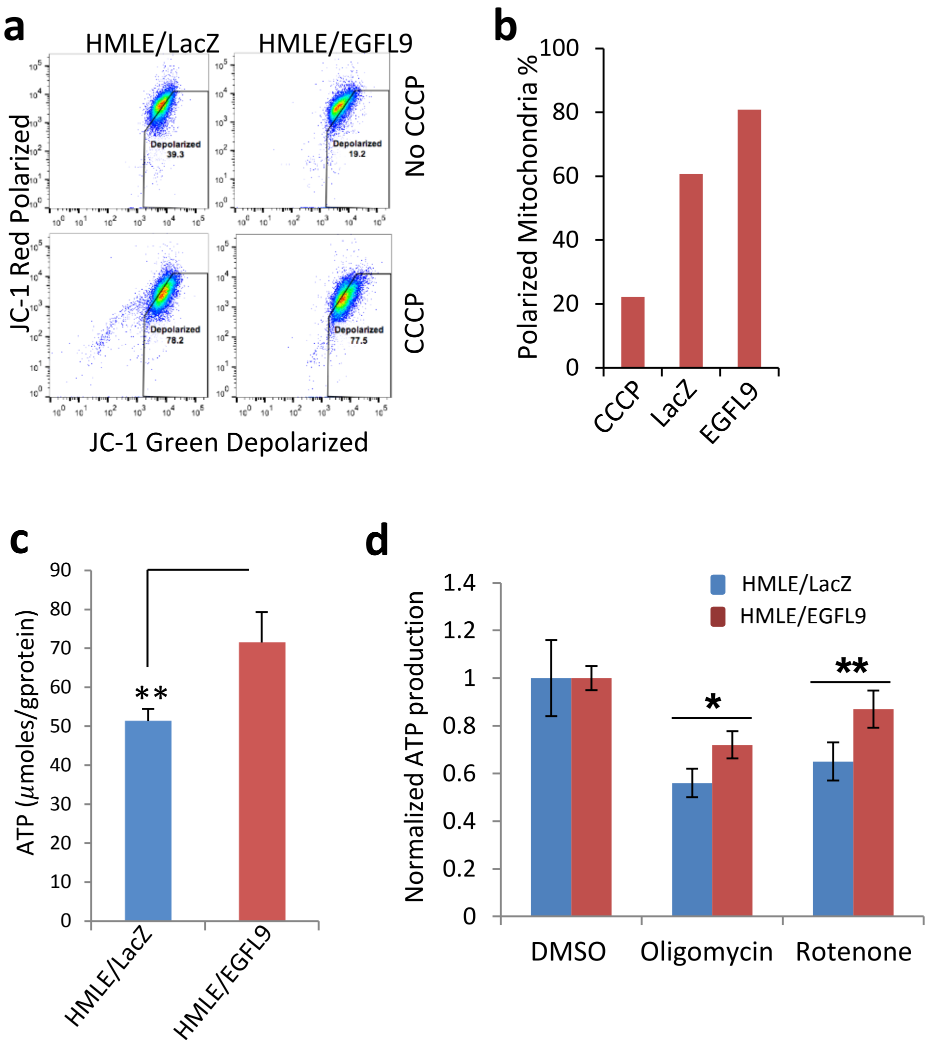

Supplementary Figure 11. Measurement of mitochondria membrane potential (MMPT) and ATP generation in HMLE/EGFL9 cells. (a) Mitochondria membrane potential was examined with JC-1 staining and cells were analyzed with FACS. CCCP was used as a control. (b) A summary of MMPT polarization of HMLE/LacZ and HMLE/EGFL9 cells. (c) ATP production in HMLE/LacZ and HMLE/EGFL9 cell models was measured using an ATP detection assay kit. (d) ATP production attributed to mitochondrial OXPHOS and glycolysis. Cells were treated with Oligomycin (10 µM) or Rotenone (10 µM) for 30 mins to inhibit OXPHOS. ATP production was then measured using the same kit as in panel c. For both c and d, Each bar represents the mean ± SD. for triplicate experiments. Unpaired two-tailed *t*-test was used for comparing two groups of data. * *P*<0.05; and ***P*<0.01.

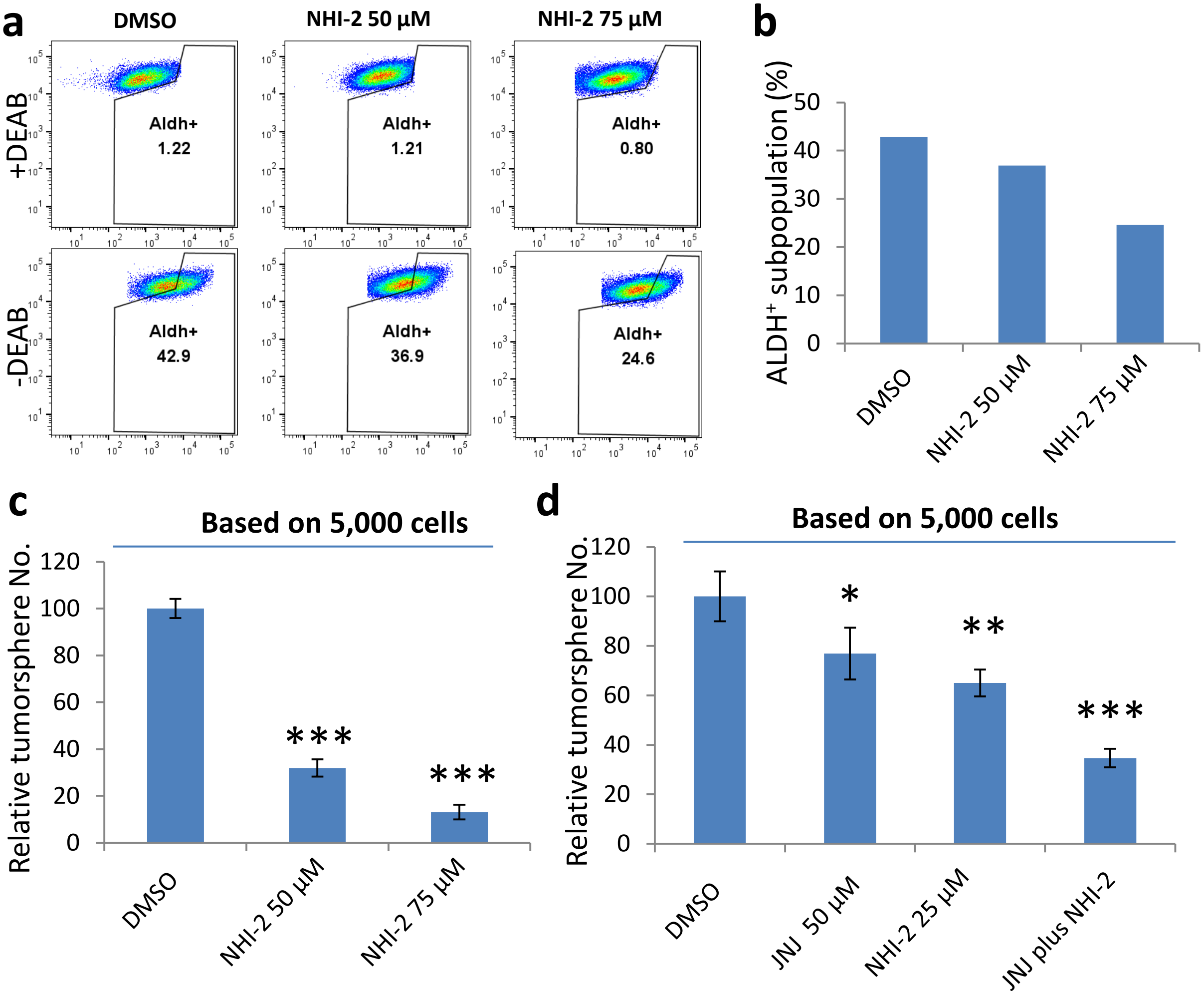

Supplementary Figure 12. The effect of glycolysis inhibitor NHI-2 on stemness of HMLE/EGFL9 cells. (a) $ALDH^+$ FACS profiles are shown for HMLE/EGFL9 cells with different doses of NHI-2 treatment. (b) Summary of percentage of $ALDH^+$ cells in HMLE cells with EGFL9 expression and 50 µM and 75 µM of NHI-2 treatment. (c) elative tumorsphere formation in HMLE/EGFL9 cells with and without different doses of NHI-2 treatment. (d) Tumorsphere formation assay of HMLE/EGFL9 (0.5×10^4) cells with JNJ38877605 (50 µM) and/or NHI-2 (25µM) treatment. For both c and d, Each bar represents the mean + SD. for triplicate experiments. Unpaired two-tailed t-test was used for comparing two groups of data. * P<0.05; **P<0.01, and ***P<0.001.

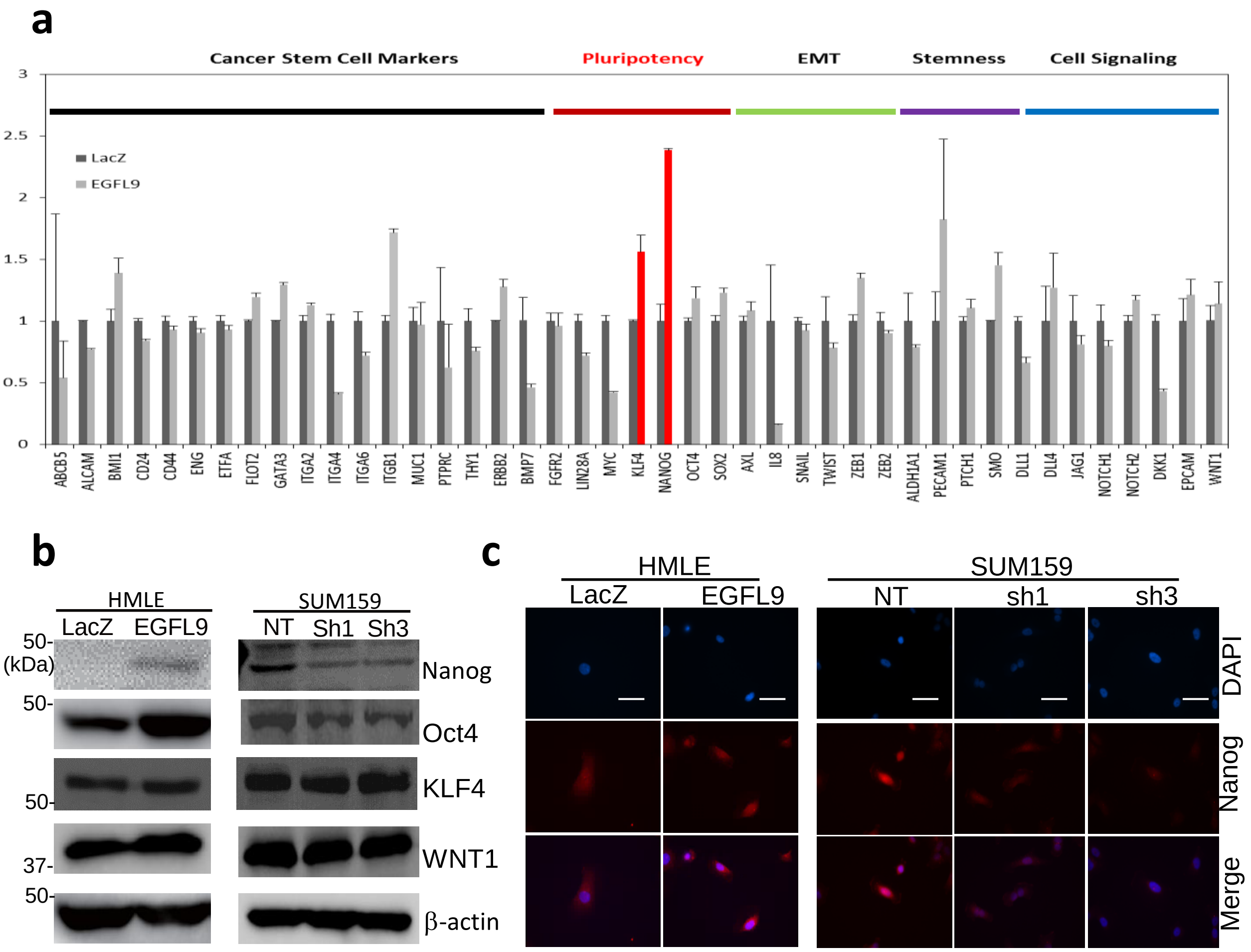

Supplementary Figure 13. Validation of EGFL9 effect on stemness-related markers. (a) The effect of EGFL9 on the expression of stem cell markers. Real-time PCR was used to determine the expression of stemness-related markers in HMLE/EGFL9 cells compared to HMLE/LacZ cells. (b) Western blotting was performed to validate the RT-PCR results. Antibodies used were as indicated. (c) Immunofluorescence analysis of Nanog expression in HMLE and SUM159 cells with ectopic expression (left panels) or knockdown (right panels) of EGFL9. Nanog staining (red signaling) is shown on top panels. Nuclei were stained with DAPI and merged images were shown on low panels. Scale bar: 100 µm.

Figure 1b

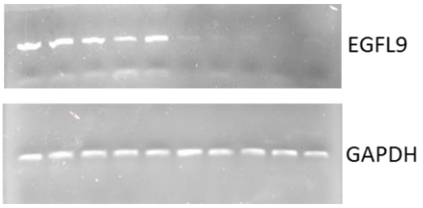

Figure 1c

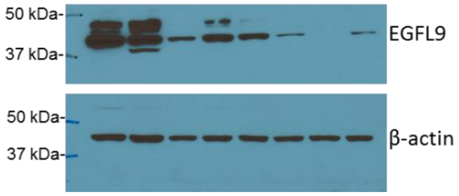

Figure 5a

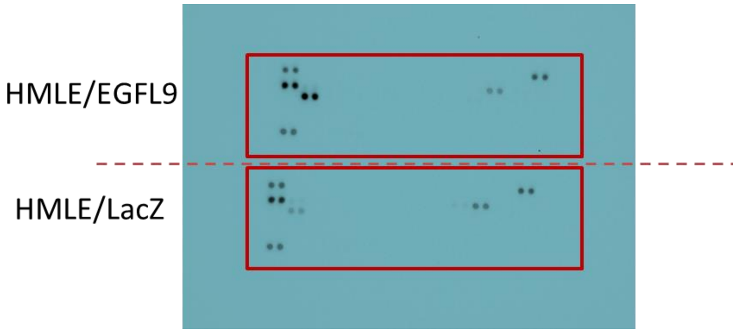

Figure 5c

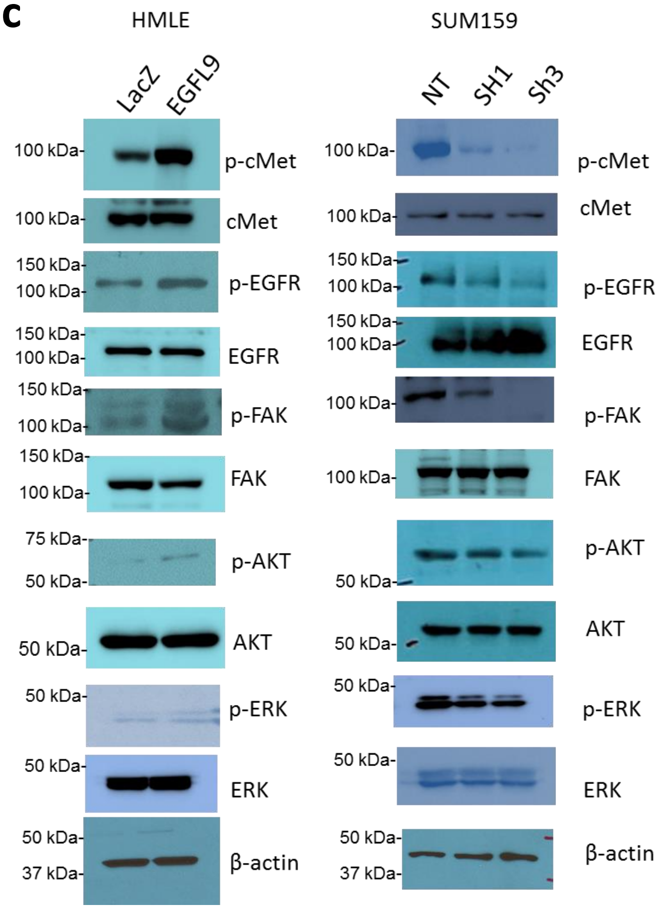

Figure 5d

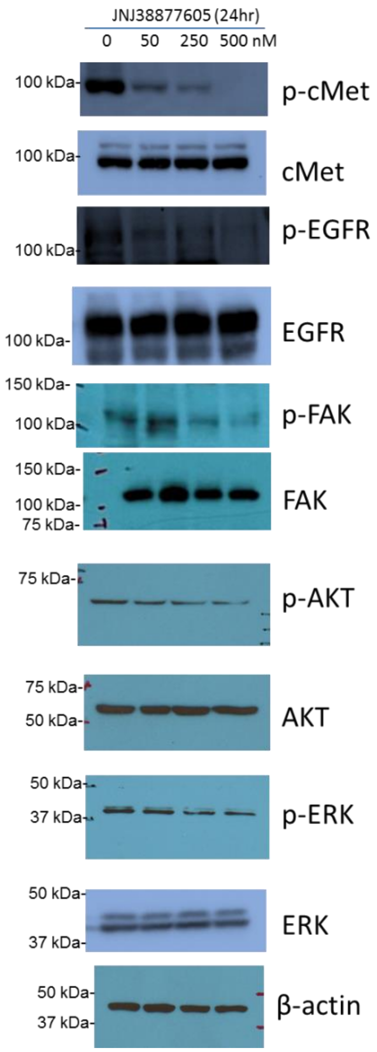

Figure 5f

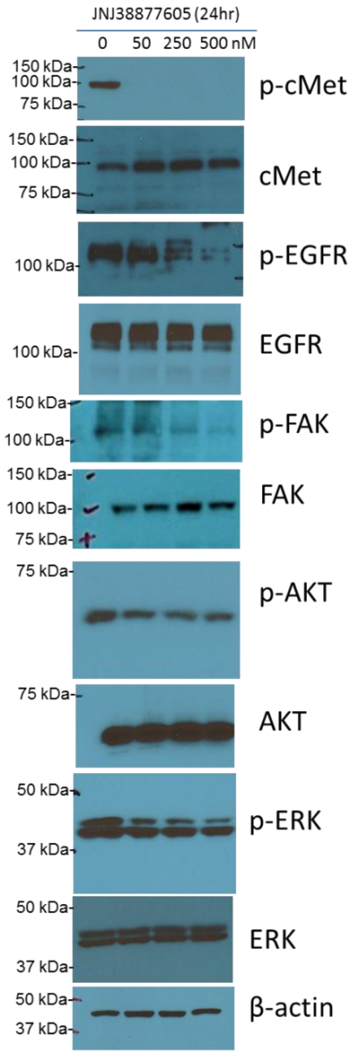

Figure 6a

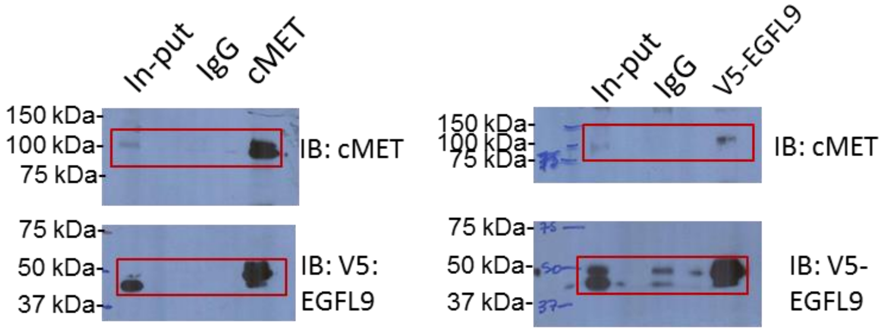

Figure 6b

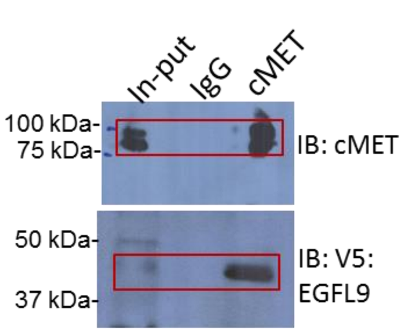

Figure 6f

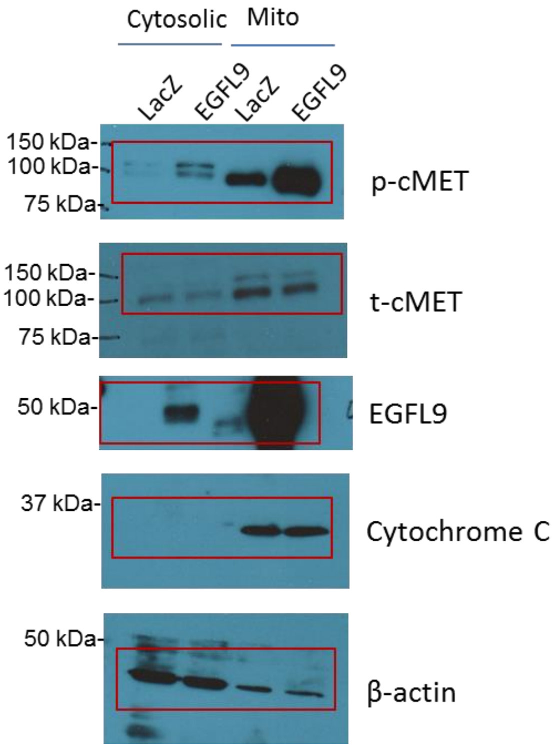

Figure 6g

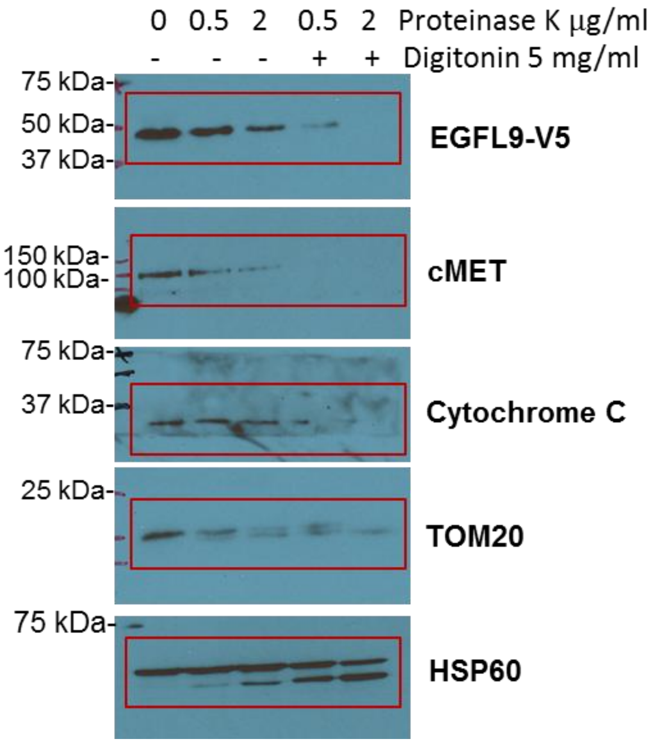

Figure 7b

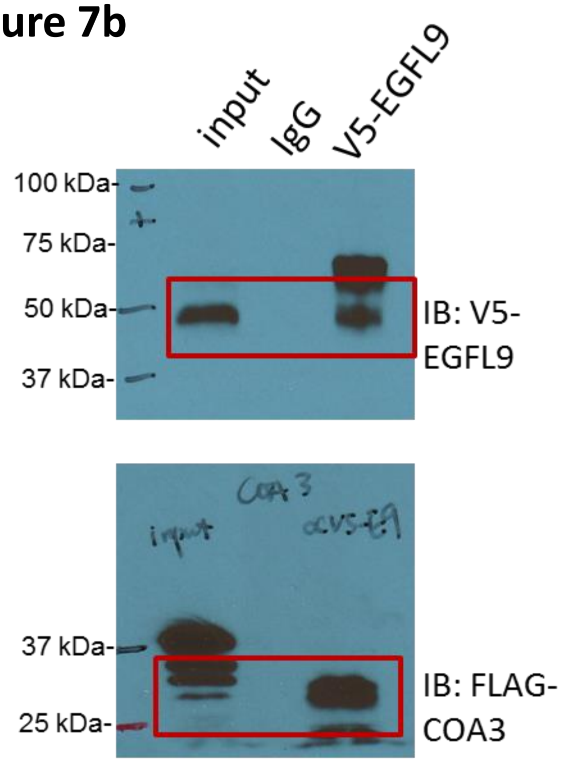

Figure 7e

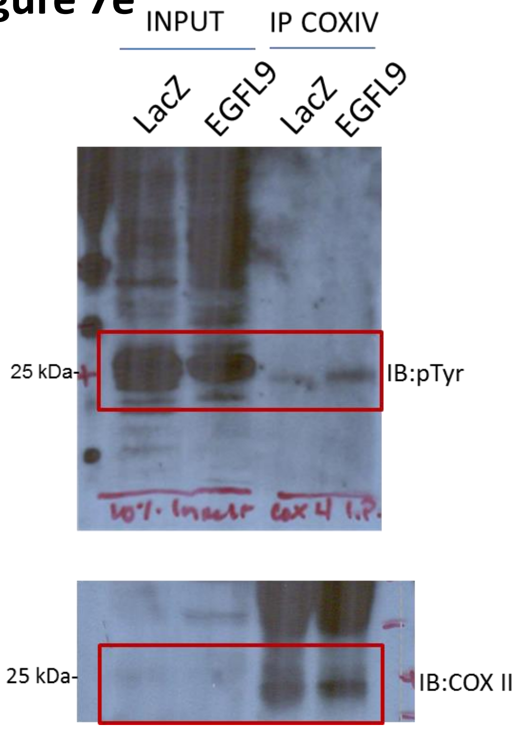

Supplementary Figure 14. Unprocessed images of blots. Uncropped images of scanned immunoblots shown in figures were provided.

Supplementary Table 1. EGFL9 primers used in cloning and Real time - qPCR

Target genes primers	Forward primers (5-'----3')	Reverse primers (5–'—3')
hEGFL9	CACCATGCCCAGCGGCTGC	CAGTGCTGTGGTCTTTCCA
mEGFL9	GATCAAGCTTGCCACCATGAAAATC ACGATGCGCGTATCAG	GATGCTCGAGTTACGTAGAATCGAGACCGAGGAGAGGG TTAGGGATAGGCTTACCCAGCGCTGTGGTCTTACCAGG
**RT-PCR primers**		
hEGFL1	CAGCTTACTATGGCCCATCC	AGGTACCATGGCACAGACAC
hEGFL2	CGTCGAACCTCTGGCTCTACA	CCAAACCCTCAGGCCATCGT
hEGFL3	GCTCACAATCACTCGGCATC	GGCTTCTACGGACACAATGC
hEGFL4	TGCCATCAATGGGTGTCAGG	GTGAGTACCGACCACTGGGA
hEGFL5	AGCAGTGACATCTACAGGCAG	TGCAGTTCGGGCCTATGTAA
hEGFL6	GCGAGGAGATGTGTTTTTCCC	AGCGCTTTCCTTTGGACCAG
hEGFL7	CACCCAGAGGAGAAGGCCA	ACCAGAAGCCACATCAGCAG
hEGFL8	AAAGAAGCCAGCCTGTAGGG	CAGGAGGAAGGAGAATCCGC
hEGFL9	AGGCCAGTGCATGTATGACG	GCGGCACGTGAAGTTGAGAG
hGAPDH	TCTTTTGCGTCGCCAGCCGAG	TGACCAGGCGCCCAATACGAC
mEGFL9	GCTGGGCAGGAAAGTTCTG	TGCCCTCCATTCCGGCACG
mGAPDH	AATGGGGTGAGGCCGGTGCT	CACCCTTCAAGTGGGCCCCG

Supplementary Table 2. EGFL9 expression in breast tumor samples.

	weak/no	mediate	strong	total	P value
EGFL9 expression and AR in a TMA					
AR(+)	9	15	4	28	
AR(-)	22	14	6	42	
total	31	29	10	70	p=0.2387
EGFL9 expression and ER in a TMA					
	weak/no	mediate	strong	total	
ER(+)	15	15	4	34	
ER(-)	16	14	6	36	
total	31	29	10	70	p=0.7858
EGFL9 expression and PR in a TMA					
	weak/no	mediate	strong	total	
PR(+)	11	11	3	25	
PR(-)	20	18	7	45	
total	31	29	10	70	p=0.8594
EGFL9 expression and Her2 in a TMA					
	weak/no	mediate	strong	total	
HER(+)	19	18	6	43	
HER(-)	12	11	4	27	
total	31	29	10	70	p=0.9722
EGFL9 expression and EGFR status in a TMA					
	weak/no	mediate	strong	total	
EGFR(+)	6	5	3	14	
EGFR(-)	25	24	7	56	
total	31	29	10	70	p=0.6106
EGFL9 expression and p53 status in a TMA					
	weak/no	mediate	strong	total	
P53(+)	6	7	2	15	
P53(-)	25	22	8	55	
total	31	29	10	70	p=0.8361
EGFL9 expression and tumor grades in a TMA					
	weak/no	mediate	strong	total	
0/i	2	3	0	5	
iia	17	13	4	34	
iib	4	3	0	7	
iii	8	10	6	24	
total	31	29	10	70	p=0.0629
EGFL9 expression and metastasis in a TMA					
	weak/no	mediate	strong	total	
Met	8	10	7	25	
Non-Met	23	19	3	45	
Normal	5	0	0	5	
total	36	29	10	75	p=0.0213

Supplementary Table 3. Sequences of shRNA used for gene knockdown

Target Gene	shRNA Clone ID	Mature Antisense Sequence
mEGFL9 (sh1)	TRCN0000124454	AAGGTAGCTAGAAAGGCTCCT
mEGFL9 (sh2)	TRCN0000124455	TTCACTGAGATCCTTAGCAGG
mEGFL9 (sh3)	TRCN0000124456	TTATGCCATCAATGCATGTGG
mEGFL9 (sh4)	TRCN0000124457	ACAGGCAGTCAAAGTCATGGA
hEGFL9 (sh1)	TRCN0000055698	TTTGTCACAGAACTTGCCTGC
hEGFL9 (sh2)	TRCN0000055699	ATCCACATTTACCTCACAGCG
hEGFL9 (sh3)	TRCN0000055700	TTATGCCGTCAAGGCAGGTGG
hEGFL9 (sh4)	TRCN0000055701	TAAGCACACACAATGGTACTC
hEGFL9 (sh5)	TRCN0000055702	AAGTTGAGAGCAAAGCCCTGG

Supplementary Table 4. Real Time Q-PCR primers for stem cell markers

Target genes	Forward primers (5'---3')	Reverse primers (5'---3')
EPCAM	gaa gag aat ggc aaa gta tga g	gca cac aca ttt gta att tgt g
WNT1	acc ttc aca aca acg agg ca	cgc atc cag cac gtg cgc
DKK1	cag aag aac cac ctt gtc ttc	aca aca caa tcc tga ggc ac
SMO	cat act gcc cca gga tat ttc	cag gcg ctt ctg cag ctc
PTCH1	caa gtg atc gtg gaa gcc ac	ctg agt cca ggt ggg gct
DLL1	cag tcg gtg tac gtc ata tc	cgg gag tct tgc cat ctc a
DLL4	gag aag aaa gtg gac agg tg	tgc ccg tga atc cag gac g
JAG1	cct tca gcg aac aat gaa ata c	cat cac gtt tac taa caa gat c
Notch1	tgc atg cgg ctg tgt ctg c	cag tgg cgt cgt gcc atc
Notch2	gcg gat gtg aat gca gtg g	gtt gtc ctg cat gtc tcg gt
Nonog	gcc gaa gaa tag caa tgg tg	gga agg ttc cca gtc ggg
Oct4	cga aaccca cac tgc agc aga tca	acc aca ctc gga cca cat gct
Sox2	ccc cgg cgg caa tag cat gg	tcc atgcgc tgg ttc acg cc
cMYC	gga aaa cca gca gcc tcc cgc	acg gct gca ccg agt cgt ag
KLF4	cct aca caa aga gtt ccc atc	cct ggt cag ttc atc tga agc
LIN28A	ggc ggc caa aag gaa aga g	gtg gca ctt ctt ggg ctg g
DNMT1	gga gat caa gct ttg tat gtt g	ggt gcc aga aac acc cct g
BMP7	ctc cta cat gaa cgc cac c	ctg tca tcg aag tag agg ac
BMI1	tga tgt cat gta tga gga gg	gaa ctc tgt att tca atg gaa g
CD44	tgg cct tgg ctt tga ttc ttg	ggc ttt ctg tcc tcc aca g
CD24	cag cgg ttc tcc aag cac cc	gcc acc att gct ctg ccc atg t
ABCB5	cta ttg caa ggg ctc ttc tc	caa ccc gtc ctg gct tta tc
ALCAM	ctg aag cct aa gaga gaa ac	ctt gaa cca tga att gct gtc
ALDH1A1	gtg gcc gtg ata cag ttc g	gga cag cag gat gac cag
ENG	cct gct cac tgc tgc act c	ctg tgg ttg gtg ctg ctg c
ETFA	gga tga aaa gac agc aag ac	ctc agt cat ttc agg aac tac
FLOT2	cct gcc tct gtg cat gcc	cgt tgt tct gtg gga tta aaa c
GATA3	ggg acc ctg tct gca atg	gca ctt ttt gga ttt gct aga
ITGA2	gaa agc cga agt acc aac ag	gtc tca tca atc tca tct gg
ITGA4	cta ctt gga ctt att gta ctt c	tgt tga tat aac tcc aac tgt c
ITGA6	ctc gct ggg atc ttg atg c	ctc ttc ttt ccg gat cct tac
ITGB1	gca tta ctg ctg ata tgg aag	gga ttg acc aca gtt gtt acg
MUC1	ccc cct agc agt acc gat	ctc agc ggg cga cgt gc
PECAM1	agt gta cag tga agt ccg g	gga tgt cct tcc agg gat g
PROM1	caca at cct gtt atg aca agc	cttgta gac cca gaa act ac
PTPRC	gga aac aga aga ggt agt gg	gta ggt gct ggc aat gac g
THY1	gac gag ggc acc tac acg	gtg ttc tga gcc agc agg
