# Supplementary material for: EGFL9 promotes breast cancer metastasis by inducing cMET activation and metabolic reprogramming
Source: Nat Commun. 2019 Nov 6;10:5033. doi: 10.1038/s41467-019-13034-3 (PMC6834558; doi:10.1038/s41467-019-13034-3)
Supplement: Supplementary file 3 — Description of Additional Supplementary Files [file 41467_2019_13034_MOESM3_ESM.pdf]

Description of Additional Supplementary Files

File Name: Supplementary Movie 1

Description: Colocalization of EGFL9 and cMET in human mammary epithelial cells. HMLE/EGFL9 cells were cultured on the cover slide in 6-well plate. For immunofluorescence analysis, EGFL9 was probed with EGFL9 primary antibody and detected with Alexa Fluor 488-conjugated secondary antibody (green). cMET was probed with cMET primary antibody and detected with Alexa Fluor 596-conjugated secondary antibody (red). Nucleus were stained with DAPI. Fluorescence images were obtained using a Zeiss LSM510 confocal microscope. The movie was made with the Volocity (6.3.1) high performance 3D imaging software.

File Name: Supplementary Movie 2

Description: Colocalization of EGFL9 and cMET in mitochondria. HMLE/EGFL9 cells were cultured on the cover slide in 6-well plate. For immunofluorescence analysis, EGFL9 was probed with primary antibody and detected with Alexa Fluor 488-conjugated secondary antibody (green), cMET was probed with and detected with Alexa Fluor 596-conjugated secondary antibody (red). Mitochondria were stained with Mitotracker (deep red). Nucleus were stained with DAPI. Fluorescence images were obtained using a Zeiss LSM510 confocal microscope. The movie was made with the Volocity (6.3.1) high performance 3D imaging software.

File Name: Supplementary Movie 3

Description: Detection of interaction of EGFL9 and COA3 proteins with a BiFC assay. p3xFlag-EGFL9-VN and p3xFlag-COA3-VC plasmids were co-transfected into 293T cells. Forty-eight hours after transfection, live cell imaging was performed using a Zeiss LSM 510 confocal microscope. The movie was made with the Volocity (6.3.1) high performance 3D imaging software.
