# Supplementary material for: EGFL9 promotes breast cancer metastasis by inducing cMET activation and metabolic reprogramming
Source: Nat Commun. 2019 Nov 6;10:5033. doi: 10.1038/s41467-019-13034-3 (PMC6834558; doi:10.1038/s41467-019-13034-3)
Supplement: Supplementary file 7 — Reporting Summary [file 41467_2019_13034_MOESM7_ESM.pdf]

nature research

Corresponding author(s): Guojun Wu

Last updated by author(s): Oct 9, 2019

Reporting Summary

Nature Research wishes to improve the reproducibility of the work that we publish. This form provides structure for consistency and transparency in reporting. For further information on Nature Research policies, see Authors & Referees and the Editorial Policy Checklist.

Statistics

For all statistical analyses, confirm that the following items are present in the figure legend, table legend, main text, or Methods section.

n/a	Confirmed	
☐	☒	The exact sample size (*n*) for each experimental group/condition, given as a discrete number and unit of measurement
☐	☒	A statement on whether measurements were taken from distinct samples or whether the same sample was measured repeatedly
☐	☒	The statistical test(s) used AND whether they are one- or two-sided<br>*Only common tests should be described solely by name; describe more complex techniques in the Methods section.*
☒	☐	A description of all covariates tested
☒	☐	A description of any assumptions or corrections, such as tests of normality and adjustment for multiple comparisons
☐	☒	A full description of the statistical parameters including central tendency (e.g. means) or other basic estimates (e.g. regression coefficient) AND variation (e.g. standard deviation) or associated estimates of uncertainty (e.g. confidence intervals)
☐	☒	For null hypothesis testing, the test statistic (e.g. *F*, *t*, *r*) with confidence intervals, effect sizes, degrees of freedom and *P* value noted<br>*Give P values as exact values whenever suitable.*
☒	☐	For Bayesian analysis, information on the choice of priors and Markov chain Monte Carlo settings
☒	☐	For hierarchical and complex designs, identification of the appropriate level for tests and full reporting of outcomes
☐	☒	Estimates of effect sizes (e.g. Cohen's *d*, Pearson's *r*), indicating how they were calculated

Our web collection on statistics for biologists contains articles on many of the points above.

Software and code

Policy information about availability of computer code

Data collection	Immunofluorescent staining image and BiFC imaging were collected using a Zeiss LSM 510 confocal microscope; RTK array and western blot images were collected using Bio-Rad Molecular imager Gel Doc XR system; Flow Cytometry results were collected using a BD LSR II flow cytometry; Q-PCR was performed using a Applied Biosystems StepOne Plus ststem; In vivo MRI was performed using a 7T 60-nm vertical-bore micro-imaging system.
Data analysis	Protein colocalization and confocal z-stacks of immune-labeled section were analyzed using Volocity (6.3.1) high performance 3D imaging software; Western blot and RTK images were analysed using Image J software. Flow Cytometry results were analyzed using FlowJo software;Q-RT-PCR was performed using GraphPad Prism software; In vivo MRI results were analyzed using the imaging workstation affiliated to the imaging system.

For manuscripts utilizing custom algorithms or software that are central to the research but not yet described in published literature, software must be made available to editors/reviewers. We strongly encourage code deposition in a community repository (e.g. GitHub). See the Nature Research guidelines for submitting code & software for further information.

Data

Policy information about availability of data

All manuscripts must include a data availability statement. This statement should provide the following information, where applicable:

- Accession codes, unique identifiers, or web links for publicly available datasets
- A list of figures that have associated raw data
- A description of any restrictions on data availability

The source data underlying Figures 1a, 2a, 2b, 2c, 2d, 2e, 2f, 2g, 2h, 3a, 3b, 3c, 4c, 4e, 4f, 5e, 7c, 7d, 7f, 7g, 7h, 7i, 8e, 8f, 8h, 8i, 8j, 8k and Supplementary Figures. 1, 2e, 2g, 8d, 8e, 10c, 10d, 10e, 11c, 11d, 12c, 12dh, 13a are provided as a Source Data file. Unprocessed original scans of blots are shown in Supplementary Figure 14.

The remaining data are contained within the article, Supplementary Information or available from the authors upon request. A reporting summary for this article is available as a Supplementary Information file.

Field-specific reporting

Please select the one below that is the best fit for your research. If you are not sure, read the appropriate sections before making your selection.

☒ Life sciences ☐ Behavioural & social sciences ☐ Ecological, evolutionary & environmental sciences

For a reference copy of the document with all sections, see nature.com/documents/nr-reporting-summary-flat.pdf

Life sciences study design

All studies must disclose on these points even when the disclosure is negative.

Sample size	A two-sided independent t-test without equal variance assumption was performed to analyze the results of cell growth, colony formation, cell migration and invasion, tumor burdens and tumor metastasis results. Two-sided Cochran-Armitage trend test was used to assess linear trend in proportions of binary conditions across the ordinal level of EGFL9 IHC intensities from TMA analysis. The proportions were estimated with Wilson's 95% CI. For the dose-outcome plots for Figure 8 H through K: two way ANOVA followed by multiple comparisons was used to compare the two values at a given concentration. P value less than 0.05 is considered significant. R version 3.4.3 was used.
Data exclusions	There was no any inclusion/exclusion criteria.
Replication	Date represent the mean+SD of three times of independent experiments,*P<0.05; ** P<0.01; *** P<0.001.
Randomization	For tumorigenesis and metastasis analysis, 10 mice were randomly divided into 2 groups, 5 in each group for tumor cell implantation.
Blinding	The EGFL9 expression in tissue array was scored blindly and independently by two scientists.

Reporting for specific materials, systems and methods

We require information from authors about some types of materials, experimental systems and methods used in many studies. Here, indicate whether each material, system or method listed is relevant to your study. If you are not sure if a list item applies to your research, read the appropriate section before selecting a response.

Materials & experimental systems

n/a	Involved in the study	
☐	☒	Antibodies
☐	☒	Eukaryotic cell lines
☒	☐	Palaeontology
☐	☒	Animals and other organisms
☒	☐	Human research participants
☒	☐	Clinical data

Methods

n/a	Involved in the study	
☒	☐	ChIP-seq
☐	☒	Flow cytometry
☒	☐	MRI-based neuroimaging

Antibodies

Antibodies used	Detailed antibody information including vendor, catalog number and clone name was described in the Methods.
Validation	All antibodies were validated by the manufacturer.

Eukaryotic cell lines

Policy information about cell lines

Cell line source(s)	All human breast cancer cell lines were obtained from and characterized by cytogenetic analysis by American Type Culture Collection (ATCC, Manassas, VA). All cell lines were grown according to ATCC recommendations. Mouse cell line 4T1 is Karmanos' own property. The mouse mammary epithelial cell line EpRas and the human mammary epithelial cell line HMLE were obtained from Dr. Robert A. Weinberg's laboratory at MIT.
Authentication	All of the cell lines were authenticated upon receipt by comparing them to the original morphological and growth characteristics.
Mycoplasma contamination	All of the cell lines were were confirmed to be mycoplasma-free. Only mycoplasma-nagative cells were used for research.

Commonly misidentified lines (See ICLAC register)	No commonly misidentified cell lines were sued for this study.

Animals and other organisms

Policy information about studies involving animals; ARRIVE guidelines recommended for reporting animal research

Laboratory animals	Five-week old, female NCR Nu/Nu mice were purchased from Taconics (Hudson, NY). Five-week old, female BALB/C mice were purchased from The Jackson Laboratory (Bar Harbor, MA).
Wild animals	No wild animal was used for this study.
Field-collected samples	This study did not involve sample collected from the field.
Ethics oversight	All animal handling and procedures were approved by Wayne State University Institutional Animal Care and Use Committee (IACUC, 15-12-026).

Note that full information on the approval of the study protocol must also be provided in the manuscript.

Flow Cytometry

Plots

Confirm that:

☒ The axis labels state the marker and fluorochrome used (e.g. CD4-FITC).

☒ The axis scales are clearly visible. Include numbers along axes only for bottom left plot of group (a 'group' is an analysis of identical markers).

☒ All plots are contour plots with outliers or pseudocolor plots.

☒ A numerical value for number of cells or percentage (with statistics) is provided.

Methodology

Sample preparation	The ALDEFLUOR kit (StemCell Technologies, Durham, NC, USA) was used to isolate the cell subpopulation with ALDH enzymatic activity. Fresh cells were suspended in ALDEFLUOR assay buffer containing ALDH substrate (BAAA, 1 µM/L per 1 X 106 cells) and incubated during 40 minutes at 37°C. An aliquot of each sample of cells was treated with 50 mM/L diethylaminobenzaldehyde (DEAB), a specific ALDH inhibitor, to establish the baseline fluorescence.
Instrument	All the cell samples were analyzed by BD LSR II flow cytometry.
Software	FlowJo software was used for analyzing the results.
Cell population abundance	We analyze the ALDH+ subpopulation in HMLE cells with and without EGFL9 overexpression. We also measured ALDH+ subpopulation in HMLE/EGFL9 cells w/wo cMET inhibition.
Gating strategy	Flow cytometry was performed using a BD LSR II (BD Biosciences, San Jose, CA). Aldefluor fluorescence was detected through a 530/30 bandpass filter after excitation at 488 nm. Just prior to acquisition 10 µL of 1 µg/mL 4',6-diamidino-2-phenylindole (DAPI) solution was added as a viability dye, detected with a 450/50 bandpass and 406 nm excitation. BD FACS Diva software was used to acquire data, calculate compensation, and export FCS files. BD FACSDiva CS&T Research Beads (BD Biosciences, 655051) were used for instrument QC, and forward scatter area scaling factor was adjusted using cells.

☒ Tick this box to confirm that a figure exemplifying the gating strategy is provided in the Supplementary Information.
